# Genomic imprinting and its effects on postnatal growth and adult metabolism

**DOI:** 10.1007/s00018-019-03197-z

**Published:** 2019-07-03

**Authors:** Steven J. Millership, Mathew Van de Pette, Dominic J. Withers

**Affiliations:** 1 MRC London Institute of Medical Sciences, Du Cane Road, London, W12 0NN UK; 2 Institute of Clinical Sciences, Faculty of Medicine, Imperial College London, Du Cane Road, London, W12 0NN UK

**Keywords:** Metabolic programming, Mouse models, Maternal care, Environment, Diet, Obesity

## Abstract

Imprinted genes display parent-of-origin-specific expression with this epigenetic system of regulation found exclusively in therian mammals. Historically, defined imprinted gene functions were almost solely focused on pregnancy and the influence on the growth parameters of the developing embryo and placenta. More recently, a number of postnatal functions have been identified which converge on resource allocation, both for animals in the nest and in adults. While many of the prenatal functions of imprinted genes that have so far been described adhere to the “parental conflict” hypothesis, no clear picture has yet emerged on the functional role of imprints on postnatal metabolism. As these roles are uncovered, interest in the potential for these genes to influence postnatal metabolism and associated adult-onset disease outcomes when dysregulated has gathered pace. Here, we review the published data on imprinted genes and their influence on postnatal metabolism, starting in the nest, and then progressing through to adulthood. When observing the functional effects of these genes on adult metabolism, we must always be careful to acknowledge the influence both of direct expression in the relevant metabolic tissue, but also indirect metabolic programming effects caused by their modulation of both in utero and postnatal growth trajectories.

## Introduction

Genomic imprinting is an epigenetic phenomenon resulting in parent-of-origin-specific gene expression [1–3] in therian mammals. Pioneering experiments using parthenogenetic and androgenetic embryos demonstrated an absolute requirement for both of the parentally inherited genomes, implicating a genetic imbalance between the two and paving the way for the subsequent discovery of imprinted genes. Currently, around 150 genes have been demonstrated as imprinted and they show a large degree of overlap between mice and humans. Many of these are found in genomic clusters, each with an epigenetically regulated imprinting control region (ICR) [4, 5] that is, in effect, a differentially methylated region (DMR). Epigenetic marks including DNA methylation (at CpG dinucleotides, often found in clusters or CpG ‘islands’) and histone tail modifications (found at promoters or gene bodies), are initiated in the germline during development and maintained in the developing embryo and throughout postnatal lifespan. Most imprinted loci have been demonstrated to possess multiple layers of epigenetic control allowing appropriate expression of the active, but not silent allele. These mechanisms of regulation are beyond the scope of this article and have been extensively reviewed previously [6–9].

The development and subsequent expansion of the imprinting system may have evolved as a consequence of ‘parental conflict’ in response to selective pressures uniquely encountered by a pregnancy. This hypothesis [10] suggests a difference in the interests of the maternal and paternal genome, whereby females, who are related to all of her offspring, aim to conserve maternal resources over multiple litters and pups. In contrast, with possible multiple paternity across litters, and therefore as it is not necessarily related to other offspring of the mother, the opportunistic paternal genome seeks to extract maximal maternal resources. In keeping with this hypothesis, paternally expressed genes (PEGs) are generally growth promoting, whereas maternally expressed genes (MEGs) are growth restricting [11]. A second hypothesis, the co-adaptation model of genetic symbiosis between mother and offspring, has also been proposed [12]. This suggests that monoallelic expression of imprinted genes arose to achieve maximal efficiency of offspring development, via maternal care inputs and also directly in the offspring, and is discussed later in more detail. However, as our knowledge of imprinted gene function has expanded, we have observed an increasing number of “outlier” imprinted genes which do not obviously fit within these expected patterns, suggesting that other evolutionary pressures beyond conflicting parental genomes or co-adaptation have led to the broadening of this method of gene regulation. Interestingly, this method of gene dosage control is not exclusively a mammalian phenomenon, with evolutionary isolated systems having been observed in plants and possibly birds [13, 14]. Alongside X-chromosome inactivation, a distinct form of regulating appropriate dosage from the sex chromosomes in mammals, these methods of epigenetic regulation, whether silencing an allele or chromosome, presumably provide an advantageous phenotype, regardless of any perceived dangers associated with functionally possessing monoallelic rather than biallelic genes, i.e. masking of deleterious mutations.

Monoallelic imprinted genes are therefore subject to vulnerability in terms of gene dosage not normally encountered by non-imprinted loci, highlighted by the fact that altered imprinted gene expression is associated with a wide range of human disorders that can be modelled with specific gene modifications in mice. Prader–Willi, Angelman, Silver–Russell, Beckwith–Wiedemann syndromes and transient neonatal diabetes all result from abnormalities in the expression of specific imprinted genes (reviewed in [15, 16]). With the likely exception of Angelman syndrome, believed to be caused exclusively by the loss of expression of the *UBE3A* gene, many of these so-called imprinting disorders arise as a result of altered expression of a cluster of genes sharing the same epigenetic control, with consequent loss of expression or increased dosage of affected genes. Consistent with the roles of imprinted genes in key processes such as growth, metabolism and behaviour, these disorders result in a range of clinical features including aberrant pre- and/or postnatal growth, abnormal feeding behaviour, learning difficulties and metabolic complications [16]. Discovery of these genetic modifications, and the conditions associated with them, has led to the necessary task of picking apart the individual contributions of various genes in these clusters, made more difficult by the intrinsic epigenetic mechanisms in place regulating imprinted (and non-imprinted) genes across the same genomic region. Even so, the clinical features described in patients with these conditions are generally consistent with those observed in mouse models.

Although imprinted genes display a variety of cellular roles (cell cycle control, ion channels, protein synthesis and degradation and nutrient transport), their expression frequently shares common features of having maximal levels during the prenatal and/or postnatal period, and occurring predominantly in tissues governing resource allocation (brain, placenta, adipose tissue and pancreatic beta cells). It is, therefore, not surprising that a substantial number of imprinted genes are critical for placental function and normal fetal growth and development [17–20]. These roles extend to a wide range of processes vital for survival and development postnatally, including thermoregulation, feeding behaviour and regulation of glucose and lipid metabolism [21–31].

To understand these diverse roles, a number of different approaches have been employed to study imprinted genes and their function. Standard constitutive deletion models provide key insights into gene function and have been critical for identifying intrinsic roles of imprinted genes in regulating metabolism. However, they are limited by the nature of imprinted genes, whereby heterozygous mutations present as functionally null and are frequently associated with a wide range of both developmental and adult pathologies, and with at least some degree of mortality. In a distinct approach from more standard gene deletion models that target the coding region of a gene, a number of mouse models have been generated whereby the ICR, where differential methylation is normally found, is deleted. These deletions have often been designed to mimic those changes seen in human disease, with the resulting absence of epigenetic marks modulating the expression of some or all the genes within an imprinted cluster, producing both loss of expression and loss of imprinting (biallelic expression) within the affected cluster [32, 33]. Transgenic mouse lines, whereby additional copies of imprinted genes are expressed from a vector such as a bacterial artificial chromosome (BAC), allow the consequences of elevating imprinted gene dosage to be studied. Thus, modeling ‘loss of imprinting’ can frequently be used to elegantly contrast with loss of expression observations. Limitations to this approach centre on the already discussed complexities associated with multiple layers of epigenetic control and the chromosomal clustering of these genes. As a result, expression from these transgenes often only loosely recapitulates endogenous expression, due in part to missing distant regulatory elements on the vector, and altered expression of some, but not all genes found within an imprinted cluster. Furthermore, it should be noted that monoallelic expression and tissue distribution of certain imprinted genes, i.e. *Grb10*/*GRB10* are not always similarly regulated in mice and humans [34–38]. That being said, these genetic approaches in mice have been instrumental in identifying roles for a number of imprinted clusters and are generally at their most persuasive when used in conjunction with one another, to provide a more complete picture of specific gene function within an imprinted cluster.

As previously mentioned, perhaps the most prominent feature of imprinted genes is their relative enrichment of expression in fetal and placental tissue and their potency for modulating in utero growth potential. Abnormal growth and development in these early prenatal and/or postnatal periods due to genetic (imprinted gene mis-expression) or environmental causes (such as reduced or excessive nutrient availability) are itself strongly associated with the risk of developing metabolic disease in later life [39, 40]. This phenomenon is both interesting in a biological sense and also strongly influences how we assess adult metabolic health. However, it adds another layer of complexity to studying the function of imprinted genes and their contribution to the effects observed upon their deletion. Without the use of inducible imprinted gene deletion, it is difficult to distinguish whether metabolic phenotypes arising in adulthood are due to alterations in growth and developmental in early life, or whether the absence of imprinted gene expression is sufficient to drive metabolic phenotypes in adulthood alone. Here, we will discuss the current understanding of imprinted genes and their physiological importance, with an emphasis on their contribution to growth and metabolism at various stages of postnatal mammalian lifespan.

## Control of fetal growth and placental resources

Although this review will focus on the influence of imprinted genes on growth and metabolism after birth, it is important to at least mention the contribution of these genes in utero. A crucial structure in mammalian prenatal development, the placenta acts as a boundary between the mother and the developing fetus and is essential for hormone transport, immunity to environmental pathogens and as a source of nutrients and growth factors. A significant number of imprinted genes are expressed in the placenta itself, with the importance of their dosage in the placenta demonstrated by the finding that loss of function or loss of imprinting of several genes causes severe placental abnormalities and subsequent fetal growth defects or even lethality. For example, disruption of placental *Ascl2, Phlda2* or *Cdkn1c* results in placental structural abnormalities and perturbed placental and fetal growth [41–46]. Other imprinted genes modulate fetal growth via an effect in both placental and embryonic tissues including *Grb10* and *Dlk1* [47–51] as well as the *Igf2* and *Igf2r* system [52–54]. In this latter example, dual growth promoting effects from placenta and fetus were demonstrated by the fact that fetal growth restriction caused by placental-specific knockout of *Igf2* was worsened upon global deletion of *Igf2* [55]. These in utero interactions have been reviewed in detail [15, 20, 56] and so herein we will focus on the effects of imprinted gene expression on modulating growth and metabolism from birth onwards.

## Thriving in the neonatal nesting period

**Fig. 1:**
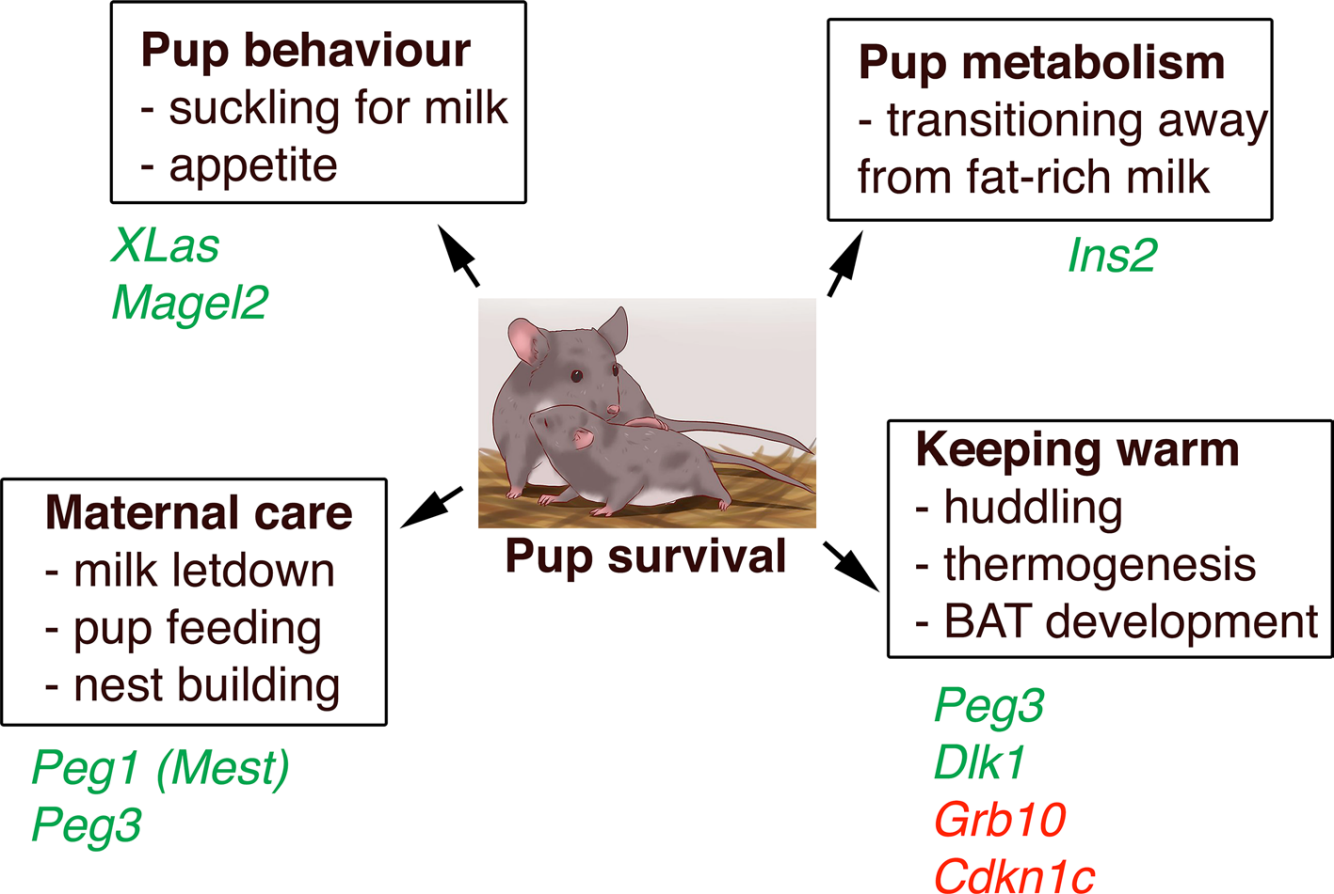
Surviving the neonatal nesting period requires multiple behavioural and metabolic inputs from both mother and pup. PEGs and MEGs that are functionally relevant to each process are displayed in green and red, respectively

Imprinted gene expression frequently extends into the neonatal and postnatal stages, regulating growth and metabolism after birth. This stage of life represents a period when there is a necessity for a combination of both behavioural actions from the pup, with its demand for nutrients (from the mother’s milk) to enable both growth and the rapid laying down of fat stores, and also to keep warm in the nest prior to full independent thermoregulation. On the other hand, maternal instincts that allow sufficient nurturing of the newborn (feeding, milk production, nest building) are of equal importance. Again, here, imprinted genes are at the centre of this crucial phase of life, and are discussed below (also see Fig. 1 for summary).

### Offspring thermoregulation and feeding behaviour

Discussed below are some of the contributions of imprinted gene function based on either metabolic or behavioural effects in the neonatal period (Fig. 1). In the pup, deletion of exon 1 at the *Gnas* locus and disruption of *XLas* in the same cluster lead to impaired suckling, resulting in high neonatal lethality [23]. Similarly, paternal disruption of *Magel2* results in reduced postnatal viability with reduced appetite and a failure to suckle [24, 57, 58]. Also intrinsic to the pup, deletion of the paternally expressed gene *Peg3* renders them chronically hypothermic and less able to defend their body temperature under cold challenge [59]. Disruption or overexpression of the paternally expressed gene *Dlk1* results in fetal and postnatal growth restriction or overgrowth, respectively, with both genetic manipulations associated with partial neonatal lethality [50, 51]. Also found at this locus, and controlled by the same shared ICR, disruption of the paternally expressed gene *Dio3* leads to partial embryonic lethality and is associated with thyrotoxicosis [60, 61]. Postnatally, disruption of the ICR at this cluster that modifies both *Dlk1* and *Dio3* dosage, amongst others, impacts upon the development of brown adipose tissue (BAT) and therefore appropriate control of thermogenesis outside of the nest and subsequent postnatal survival [26]. Huddling for warmth is a simple cooperative behaviour observed in many species, in which the costs of heat production are borne by the individual but the benefits are shared. Under the paternal conflict theory, maternally expressed genes would favour increased thermogenic contribution, while paternally expressed genes would act to restrict this process, and although some imprinted genes hold firm to this (*Grb10*, *Cdkn1c* and *Dlk1*) [26, 29, 62], exceptions to this trend have been observed (*Peg3*) [59].

### Maternal–fetal interactions and the co-adaptation model

Paternally expressed genes *Peg1* (*Mest*) and *Peg3* are strongly expressed in the developing postnatal brain and, when mutated, result in compromised maternal care in mutant nursing mothers. Specifically, this includes reduced nest building and pup care with reduced maternal food intake and impaired milk letdown [21, 22, 63]. Disruption of either of these genes in the mother culminates in reduced pup survival, even if offspring are wild type and therefore display no other growth impairments. A large number of imprinted gene deletion models adhere to the parental conflict model, whereby ablation of paternally expressed genes leads to growth defects, while ablation of maternally expressed genes leads to early overgrowth. However, although a large portion of imprinted gene functions fit with this model, there are a significant number that do not clearly adhere to these rules, and this is particularly apparent when observing maternal care paradigms.

The co-adaptation model [12] puts forward the idea that genomic imprinting arose to establish a form of genetic symbiosis between mother and offspring, where imprinted gene function acts at both the level of the mother and in offspring interaction, with silencing of one copy able to benefit offspring vigor by modulating both organisms. This occurs with the imprinted gene *Grb10*, which is unique in the sense that it is maternally expressed in peripheral tissues but paternally expressed in the CNS under a brain-specific promoter [37, 48]. Accordingly, paternal uniparental disomy (UPD) or maternal *Grb10* deletion results in prenatal and postnatal (~ 130%) overgrowth, but with a disproportionately small brain and large liver [47, 48, 64]. Additionally, these mutants display major physiological alterations in peripheral tissues including perturbed BAT-mediated thermogenesis, increased lean mass, reduced adiposity and improved glucose metabolism [37, 62, 64–66]. Inversely, paternal *Grb10* expression within the CNS appears to be more important at the behavioural level, with paternal deletion resulting in increased social dominance [37]. Gene deletion studies also demonstrate the contribution of *Grb10* expression in the mother and from the pup. Disruption of maternal *Grb10* expression in the pup appears to increase nutrient demand from the offspring, whereas maternal *Grb10* deficiency in the mother prevents any increase in milk supply from the ducts (where *Grb10* is also imprinted) under these conditions of increased demand. Therefore, *Grb10* regulates offspring growth and adiposity by mediating both sides of the mother–offspring interaction [67].

### Post-weaning catch up growth and adult metabolic complications

**Fig. 2:**
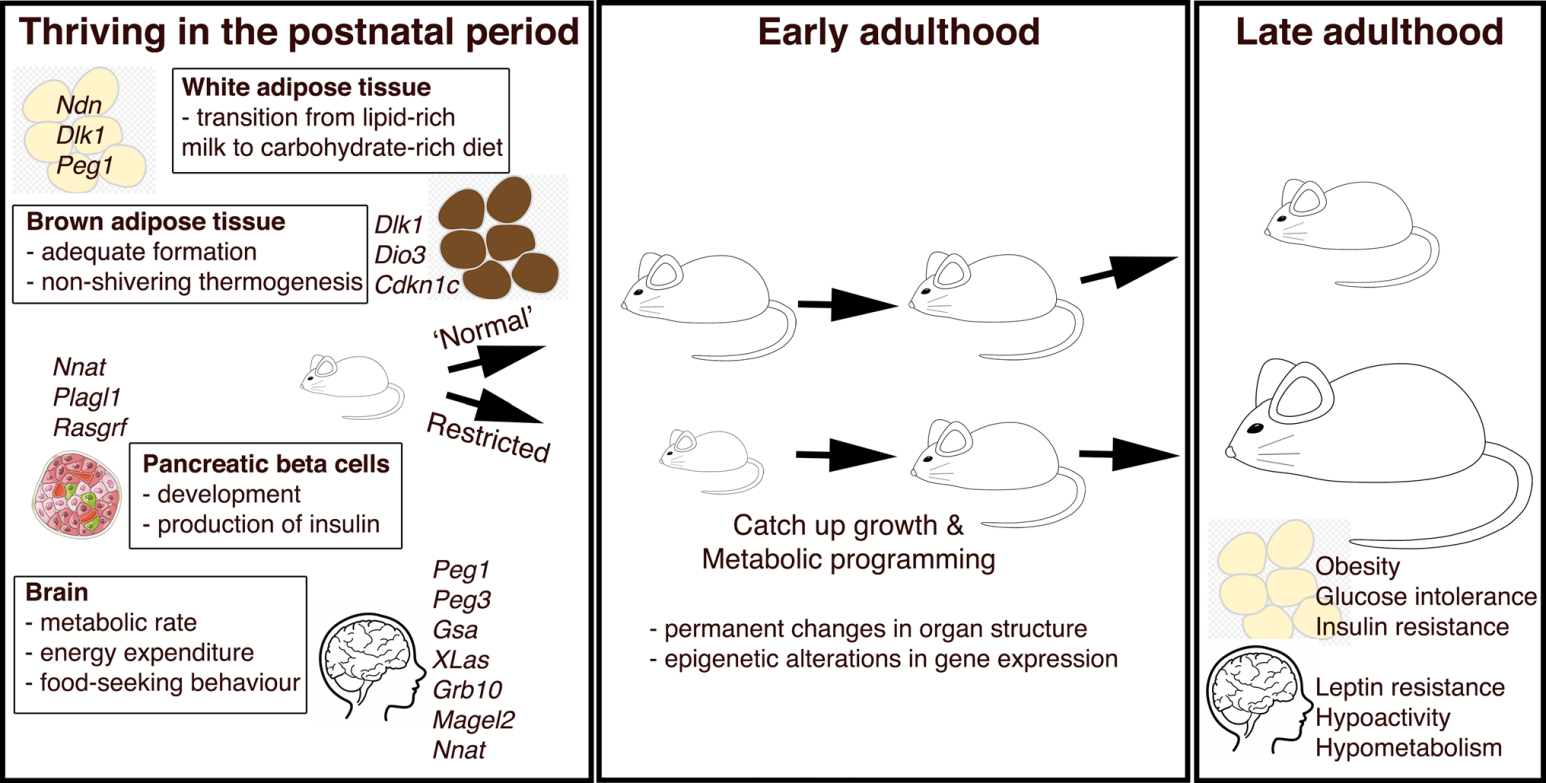
Progression to independent postnatal life requires adequate development and functionality in multiple metabolic systems. Furthermore, restricting growth and development at this crucial early stage results in metabolic programming and thus metabolic complications in adulthood

The transition to independent life involves crucial physiological requirements such as maintaining core body temperature outside of the nest, as described above. This postnatal period also requires the offspring to begin to acquire food other than that provided by their mother, with concomitant impacts upon the regulation of their metabolism. This requires a major metabolic shift, as the pup transfers from a lipid-rich milk-based source of nutrients from its mother, to a carbohydrate-rich diet [26, 68]. This shift from a generally lipolytic metabolism to a lipogenic one coincides with the deposition of white adipose tissue (WAT) in the postnatal period (Fig. 2). Insulin, a key anabolic hormone required for sufficient adipose tissue accumulation, plays a more important role in the postnatal rather than prenatal period in mice. This is demonstrated by the fact that deletion of both major forms of insulin in rodents, *Ins2* (imprinted during the gestational period but with biallelic expression postnatally) and *Ins1* (non-imprinted), or ablating the insulin receptor *Insr*, does not cause any major growth or metabolic perturbations to glucose homeostasis in utero. However, these mutants develop rapid neonatal diabetes soon after birth [69–72].

The paternally expressed imprinted gene, *Plagl1*, is located in a genomic region associated with transient neonatal diabetes in humans, caused by either paternal UPD of chromosome 6 or duplication of this region [73]. This effect is phenocopied in mice with overexpression of *Plagl1*, recreating this neonatal hyperglycaemia observed in affected children [74]. Similarly, *Rasgrf1* is a paternally expressed gene found primarily in the postnatal brain and pancreatic beta cells. Mice null for *Rasgrf1* on the paternal allele are postnatally growth restricted which continues into adulthood. In adulthood they also present with hypoinsulinaemia from reduced beta cell mass (due to reduced beta cell neogenesis and proliferation) and, therefore, demonstrate impaired glucose tolerance [75, 76].

Abnormal growth in the postnatal period (Fig. 2), due to genetic or environmental causes (such as reduced or excessive nutrient availability), influences key processes governing feeding and energy homeostasis in early adulthood. As such, these defects are strongly associated with the risk of developing obesity and metabolic disease in late adulthood [39, 40, 77]. This occurs when a developing organism responds to substandard environmental conditions during early stage programming. Ultimately, this leads to an increased risk of developing the metabolic syndrome in later life, a phenomenon known as metabolic programming that falls within the wider phenomena of fetal programming [78, 79] (Fig. 2; Table 1). Proposed mechanisms for how this early life adversity can be detrimental to long-term metabolic health include permanent changes in organ structure and programmed and sustained epigenetic changes in gene expression [80, 81]. Altered imprinted gene dosage for various paternally expressed genes is, therefore, a risk factor for obesity in adulthood. Deficiency of *Ndn* or overexpression of *Peg1* (*Mest*) in mice causes obesity owing to increases in adipose tissue expansion [82–84]. Brain-specific deletion of *Gsa*, which controls melanocortin-mediated energy expenditure in the hypothalamus, results in hypometabolism and obesity with associated glucose intolerance and insulin resistance [25, 85–87]. There are, however, cases where failure to thrive in the postnatal period does not necessarily lead to obesity and the metabolic syndrome in adulthood. Pups with disruption of *XLas* that survive to adulthood are severely growth restricted, lean, with an increased metabolic rate and are hypersensitive to insulin, and display an associated hyperphagic phenotype [23, 28]. Similarly, although maternal duplication and, therefore, biallelic expression of *Cdkn1c* in mice phenocopies Silver–Russell Syndrome with low birth weight and neonatal hypoglycaemia, these early defects result in leanness in adults, due principally to browning of WAT. These animals are also protected from diet- and age-induced obesity, and the associated worsening of glucose tolerance and insulin sensitivity. A specific role for modulation of the brown adipocyte lineage is also implicated in *Cdkn1c* loss of function studies, with the null mice displaying a failure of BAT formation [29, 88].

**Table 1:** Modelling altered imprinted gene dosage in mice and the resulting postnatal phenotypic outcomes

Imprinted gene (alias)	Expressed allele	Proposed gene function	PN behaviour, growth and metabolism	Metabolic complications in the adult	References
*Cdkn1c*	Maternal	Control of cell cycle and cellular differentiation	Neonatal hypoglycaemia (overexpression via BAC)	Lean, increased BAT activity, improved GT and IS (overexpression via BAC). Targeted demethylation at the maternal allele results in beta cell hyperproliferation	[29, 88, 119]
*Dio3*	Paternal	Negative regulator of thyroid hormone metabolism	Pup thyrotoxicosis, partial lethality (homozygous gene disruption)	Reduced insulin secretion and GT (homozygous gene disruption)	[60, 61]
*Dlk1* (*Pref1*)	Paternal	Cellular differentiation, inhibits preadipocyte to adipocyte conversion	PN GR (gene disruption, paternal allele) or PN overgrowth (BAC overexpression), partial lethality (both gene disruption on paternal allele and via BAC overexpression)	Obese, hyperphagic, reduced EE, impaired GT and IS (gene disruption, paternal allele). Lean and improved GT and IS (BAC overexpression)	[26, 49–51, 93]
*Grb10* (*Meg1*)	Maternal	Receptor tyrosine kinase adaptor protein	Reduced maternal resource allocation, PN increased demand and overgrowth in pup (gene disruption, maternal allele)	Lean, improved GT and IS (gene disruption, maternal allele only and also homozygous disruption)	[37, 47, 48, 62, 64–67]
*Gsa*	Maternal	G-protein alpha subunit, signal transduction	Impaired pup suckling (gene disruption, maternal allele)	Obese, impaired GT and IS, reduced MR (gene disruption, maternal allele)	[25, 85–87]
*Igf2*	Paternal	Growth factor	Severe postnatal GR (gene disruption, paternal allele)	Deletion of region 5′ to ICR on either allele results in brain-specific reduction of *Igf2* and obesity with reduced FI. Transgenic overexpression results in a lean phenotype with reduced adipose lipid content	[54, 120, 121]
*Magel2*	Paternal	Ubiquitin ligase enhancer	Impaired pup suckling (gene disruption, paternal allele)	Obese, hypoactivity, LR, reduced EE, hyperinsulinaemia (gene disruption, paternal allele)	[24, 57, 58, 91, 92]
*Ndn*	Paternal	Promotes neural differentiation and survival	PWS phenotype	Obesity from gene disruption (paternal allele) or lentivirus-mediated knockdown in adipose	[84]
*Nnat* (*Peg5*)	Paternal	Signal peptidase-mediated facilitation of preprohormone translocation	PN GR (gene disruption, paternal allele)	Obese, hypoactive, reduced EE, increased FI, LR (gene disruption, paternal allele)	[30, 31]
*Peg1* (*Mest*)	Paternal	Alpha/beta hydrolase	Poor maternal care (gene disruption, paternal allele)	Lean (gene disruption, paternal allele). Transgenic overexpression in adipose results in obesity	[22, 82, 83]
*Peg3*	Paternal	Zinc finger protein, controls apoptosis	Poor maternal care, pups are cold sensitive, GR (gene disruption, paternal allele)	Obese, reduced EE and LR (gene disruption, paternal allele)	[21, 59, 63]
*Plagl1* (*Zac1*)	Paternal	Zinc finger protein and suppressor of cell growth	Transient neonatal diabetes (transgenic overexpression)	Impaired GT (transgenic overexpression)	[73, 74]
*Rasgrf1*	Paternal	Guanine nucleotide exchange factor	PN GR (gene disruption, paternal allele)	Lean, hypoinsulinaemia and impaired GT (homozygous gene disruption)	[75, 76]
*XLas*	Paternal	G-protein alpha subunit, signal transduction	Impaired pup suckling, GR (gene disruption, paternal allele)	Lean, increased MR, increased GT and IS (gene disruption, paternal allele)	[23, 28]

*BAC* bacterial artificial chromosome, *EE* energy expenditure, *FI* food intake, *GR* growth restriction, *GT* glucose tolerance, *IS* insulin sensitivity, *LR* leptin resistance, *MR* metabolic rate, *PN* postnatal, *PWS* Prader–Willi syndrome

Overall, these mouse models with altered expression of imprinted genes demonstrate a failure to thrive in the early postnatal period, mimicking the pattern observed in human imprinting disorders such as Prader–Willi and Silver–Russell syndrome [89, 90] (summarised in Table 1). A common feature of these early defects is a postnatal ‘catch up’ growth phase following this failure to thrive period. *Nnat* (*Peg5*) is not expressed in the placenta and does not alter fetal growth, but its deletion causes a postnatal growth restriction and subsequent catch up growth following weaning [30]. This catch up growth acts to re-establish the growth of an organism back to its ‘normal’ trajectory (reviewed in [39]) and is frequently found after manipulation of imprinted gene dosage in mutant mice, often in the neonatal and/or postnatal period, where mice are transitioning to leaving the nest (reviewed in [15]). In adulthood, *Nnat* null mutants have lower energy expenditure and are hypoactive, leptin resistant and hyperphagic, which together lead to the development of obesity [30]. Obesity also results from deletion of paternally expressed genes *Peg3*,* Dlk1* or *Magel2*, with differences in feeding and energy expenditure, all of which exhibit early postnatal catch up growth in the null allele of each gene [24, 51, 57, 59]. *Magel2* mutation is associated with a postnatal failure to thrive, with pups that reach weaning age developing obesity and hyperinsulinaemia, with associated reduced energy expenditure, even in the face of hypophagia and hypoactivity [57, 58, 91, 92]. Although displaying reduced survival as pups, *Peg3* null mice that survive to early adulthood are underweight and hypophagic with delayed postnatal adipose deposition, but with elevated adiposity in later life. This is due to hypothalamic dysregulation manifesting as reduced metabolic rate and core body temperature and also leptin resistance [59, 63]. Mice with altered expression of *Dlk1* have postnatal growth defects with partial lethality [49, 51]. Mutant mice surviving until weaning demonstrate postnatal catch up growth and increased adiposity on both normal and high fat diet [49, 51]. Conversely, mice with transgenic *Dlk1* overexpression, although displaying increased prenatal growth, also show partial lethality due to major organ abnormalities [50]. Surviving adults, however, have reduced adult adiposity and resistance to high fat diet, with improved glucose tolerance and insulin resistance [93].

## Imprinted gene modulation in response to the environment

Our increased understanding of imprinted genes has revealed a multitude of functions that extend beyond the in utero period, regulating growth potential, resource allocation and metabolism from an early stage embryo through to adulthood and into later life. Primary epigenetic marks, including differential DNA methylation of the ICRs that regulate imprinted genes, are laid down in the respective germlines and maintained throughout lifespan of the individual. While these marks are believed to initiate monoallelic expression, secondary imprinting marks, which can also include DNA methylation but extend to modification of histone tails and chromatin architecture, are established post-fertilisation and are thought to help retain the correct expression of the gene or cluster. It has been shown in numerous experiments that epigenetic marks can be susceptible to environmental modulation [94–101]. One could, therefore, hypothesize that imprinted genes would be environmentally sensitive as a group, due to the requirement of these epigenetic marks to ensure appropriate allelic expression. These marks are laid down in utero, either via the methylation of the ICRs in the newly formed germ cells of the embryo, or via secondary imprinting marks in the developing somatic tissues. This would, therefore, suggest that the in utero period provides a key window for environment-dependent programming of imprinted gene expression.

An early study into this potential sensitivity showed that the ICRs as a group were insensitive in offspring that had been exposed to gestational protein restriction [102]. The authors did, however, note that there were some modest changes in the expression of those imprinted genes analysed. More recently, studies have shown that the putative secondary imprinting marks may in fact show some environmental sensitivity [94]. The clearest indication of this response was demonstrated by a new approach to studying imprinted genes, where a firefly luciferase knock-in model was used to image allelic expression of the *Cdkn1c* gene. Upon gestational low protein exposure, a loss of imprinting occurred whereby exposed embryos were found to have active transcription of the *Cdkn1c* gene from both of the parentally inherited alleles, which was retained into adulthood. These changes were found to be caused by gestational erosion of differential DNA methylation of a region spanning the *Cdkn1c* promoter, a secondary imprinting mark that usually retains silencing of the paternal allele. Perhaps most interestingly, these changes could be largely buffered against by the supplementation of dietary folate, indicating that the limited availability of dietary methyl donors was the cause of these epigenetic changes [94].

While studies have shown that as a group imprinted genes show no specific responsiveness to environmental modulation [103], it remains most likely that certain imprinted loci are sensitive to specific stimuli. The reasons for this selective sensitivity, or the phenotypic consequences of the resulting expression changes, are not currently understood. However, the different mouse models of imprinted gene modulation that have so-far been discussed would suggest that any changes are likely to be profound and lifelong. A recent study using haploinsufficiency of the epigenetic modifier *Trim28* demonstrated that imprinted gene dysregulation can provide a clear indicator of metabolic fitness, and these observations appeared to be closely re-capitulated in cohorts of obese children [104]. It should also be noted that due to the unique epigenetic regulation that imprinted genes undergo, with the establishment of methylation marks in the developing germlines, any sensitivity of these marks to environmental pressures in pregnancy has the potential to directly influence both the F1 and F2 generations. However, studies concerning multi-generational and trans-generational effects on imprinted genes have, until now, provided somewhat conflicting data [105–109]. The extent of environmental sensitivity of this group of genes will only truly be established through further well-controlled studies.

## Concluding remarks

Imprinted genes function in multiple cellular pathways but their roles appear to converge on postnatal processes that modulate early growth and behaviour. Unsurprisingly, genetic deletion in mice has proved to be a major source of knowledge in terms of imprinted gene function (Table 1), although the organisation of imprinted genes into genomic clusters often makes the design or analysis of these mutant mouse models difficult. However, these null alleles have been crucial to our understanding of imprinted gene function. Transgenic models expressing more than one active allele and, therefore, modelling loss of imprinting often results in severe phenotypic outcomes and so provide evidence for the evolutionary impetus behind the reduced dosage of imprinted genes in a particular organism. Imprinted genes are expressed in the fetus and placenta during gestation and, clearly, are important for placental function and embryonic growth [1–3]. The fact that the development of genomic imprinting appears to have coincided with the appearance of the placenta in evolution strongly suggests an interrelated role for both the regulation of the supply and demands of nutrients in utero [5, 11, 110]. However, it has also been suggested that genomic imprinting arose as a result of co-adaptation between the mother and her offspring to achieve optimal fitness for both via placental resource allocation in utero and via lactation in the nesting period [12, 111].

Without information from inducible deletion mouse models, where imprinted gene expression is retained in early life and deleted in adulthood, it has been challenging to tease apart the relative contributions of imprinted genes in early life and adulthood. However, as has been described, a growing number of direct functional roles have been assigned to imprinted gene expression in adult metabolic tissues. Whether there is added evolutionary impetus for genes to be imprinted as they affect adult metabolism either directly or indirectly remains unclear. Certainly, it can be said that using current hypotheses for why imprinting arose in mammals, it is challenging to explain many of the observed effects in adult tissues of transgenic mice. It remains likely that the initial driving force for the appearance of the imprinting system arose alongside the emergence of the mammalian pregnancy, and not due to any potential benefits observed in adult tissue. That is not to say that evolutionary pressure centering on adult metabolic tissues has not been a key driving force for the subsequent expansion of the imprinting system that has occurred with the mammalian phylogenic evolution.

Failure to thrive in infancy leads to complications in adulthood (Table 1). The postnatal period requires a combination of behavioural (suckling for milk) and metabolic (independent thermoregulation) actions in the offspring as well as sufficient maternal care (feeding, milk production, nest building), with imprinted genes central to these processes. Postnatal offspring are then required to begin to seek food other than that provided by their mother and subsequently shift their internal metabolism to a lipogenic directionality, coinciding with an increase in adipose tissue stores. A failure to thrive in this crucial period, marked by phenotypic features such as postnatal growth restriction and early metabolic defects, permanently alters developmental trajectories in the offspring. These alterations in fat deposition and hypothalamic circuitry lead to metabolic complications in the adult, often in the form of obesity, as a direct result of phenotypes such as hyperphagia, reduced energy expenditure and leptin resistance. Ultimately, obesity is the cause of multiple secondary metabolic disorders in the periphery such as reduced glucose uptake and insulin resistance. However, a restriction to proper adipose expansion is itself a driver of the metabolic syndrome, as the inability to store potentially toxic lipids in inert adipose tissues leads to their deposition in more sensitive peripheral organs including muscle and liver [112].

An array of metabolic disorders stems from inappropriate expression levels of imprinted genes [85, 89, 90, 113–117]. With a greater understanding of imprinted gene function, we are now beginning to see that these effects are both indirect, through early life programming, and direct in adult tissues, with subtle modulations in expression capable of inducing profound phenotypic consequences. Imprinted genes, therefore, present as an interesting and novel target for pharmaceutical intervention in metabolic disease. Due to their well-characterised epigenetic regulation, targeted therapies have the potential to “reprogramme” desired tissues, with early studies indicating great potential for this route [118]. As more functional roles are uncovered, so the interest in this group of genes will continue.
